# Homing and Engraftment of Hematopoietic Stem Cells Following Transplantation: A Pre-Clinical Perspective

**DOI:** 10.3390/curroncol31020044

**Published:** 2024-01-23

**Authors:** Tanvir Hasan, Ajay Ratan Pasala, Dhuha Hassan, Justine Hanotaux, David S. Allan, Harinad B. Maganti

**Affiliations:** 1 Canadian Blood Services, Stem Cells and Centre for Innovation, Ottawa, ON K1G 4J5, Canada; tanvir.hasan@blood.ca (T.H.); ajay.pasala@blood.ca (A.R.P.); dhuha.hassan@blood.ca (D.H.); justine.hanotaux@etu.uca.fr (J.H.); 2 Department of Biochemistry, Microbiology and Immunology, Faculty of Medicine, University of Ottawa, Ottawa, ON K1H 8L6, Canada; 3 Clinical Epidemiology & Regenerative Medicine, Ottawa Hospital Research Institute, Ottawa, ON K1Y 4E9, Canada

**Keywords:** hematopoietic stem-cell transplantation, xeno-transplantation, homing, engraftment, clonal hematopoiesis, clonal leukemogenesis, competitive repopulation

## Abstract

Hematopoietic stem-cell (HSC) transplantation (HSCT) is used to treat various hematologic disorders. Use of genetically modified mouse models of hematopoietic cell transplantation has been critical in our fundamental understanding of HSC biology and in developing approaches for human patients. Pre-clinical studies in animal models provide insight into the journey of transplanted HSCs from infusion to engraftment in bone-marrow (BM) niches. Various signaling molecules and growth factors secreted by HSCs and the niche microenvironment play critical roles in homing and engraftment of the transplanted cells. The sustained equilibrium of these chemical and biologic factors ensures that engrafted HSCs generate healthy and durable hematopoiesis. Transplanted healthy HSCs compete with residual host cells to repopulate stem-cell niches in the marrow. Stem-cell niches, in particular, can be altered by the effects of previous treatments, aging, and the paracrine effects of leukemic cells, which create inhospitable bone-marrow niches that are unfavorable for healthy hematopoiesis. More work to understand how stem-cell niches can be restored to favor normal hematopoiesis may be key to reducing leukemic relapses following transplant.

## 1. Introduction

Hematopoietic stem-cell (HSC) transplantation (HCT) has been established as a powerful therapeutic and research tool for close to a century. HCT has been instrumental in understanding blood stem-cell biology, both in homeostasis and in disease conditions. A plethora of hematologic disorders, including various forms of leukemia, sickle cell anemia, myelodysplastic syndrome, and bone-marrow failure can be treated with HCT. While tremendous advancement has been made in the safety, efficacy, and application of HCT, challenges remain for improving this treatment method further. Preclinical animal models, and especially xenotransplant models, in which human cells can be transplanted into engineered mice that allow immune tolerance, have played a pivotal role in understanding human HSC biology. In this review we will highlight some key concepts, such as HSC homing, engraftment, and competitive repopulation, using the pre-clinical lens, to allow greater fundamental understanding of the full potential of HCT.

## 2. Xenotransplantation Models in HCT Research

In recent years mice strains like NOD scid IL2Rγ^Null^ (NSG) and NOD Rag1^Null^ IL2Rγ^Null^ (NRG) have been widely used for HCT research. Both strains are based on non-obese diabetic (NOD)/severe combined immunodeficiency (SCID) models. The NSG mice contain a mutation in the γ chain of the interleukin 2 (IL-2) receptor, rendering it non-functional, which depletes the immune system of these mice severely. These mice have been reported to be able to reconstitute a wider range of human hematopoietic cell types (T and B lymphocytes, myeloid cells, plasmacytoid dendritic cells, and NK cells), compared to the original NOD/SCID models, following transplantation of human CD34+ cells. The capacity to support higher levels of human HSC engraftment is also greater in these mice [1,2]. The NSG mice are the current gold-standard for HCT research and are widely used across the world. There are several variants of NSG mice that are used for specific research requirements. For example, mutation in the Rag1 gene in NRG mice makes them more tolerant to the effects of irradiation (and genotoxic agents), while at the same time maintaining the similar level of human cell engraftment capacity. Therefore, these mouse models can be used in cancer radiotherapy research where preservation of bone-marrow niche is important for optimum HSC engraftment [3]. Thus, the use of different mouse models is crucial in understanding different aspects of HCT process.

While the murine models are well-established and documented in the HCT research, other model animals, like zebrafish, can also provide insight into HSC function and properties. Human HSCs have been xeno-transplanted in zebrafish to interrogate the physiological as well as pathological processes involved. The transparency of the zebrafish embryos makes them a suitable choice to be used for high-resolution imaging techniques to visualize the growth and dynamics of the transplanted cells in vivo, which is not an option when using murine models [4,5]. Thus, these animal model systems have become invaluable in understanding and studying HCT.

Research use of the animal models and translation of the findings to clinical practice present some unique challenges. The physiological, immunological, and genetic landscapes are different in mice and humans, which can reduce the direct transferability of the experimental results [6,7]. For example, the evolving view of hematopoiesis, HSC, and progenitors, from the hierarchical model to the continuum model, demands more studies of the process in humans, as the current models are largely based on the studies in murine systems (which are extensively reviewed by Haas, Trumpp, and Milsom [8]). Moreover, NSG mice have a lifespan of about approximately 90 weeks [1], whereas the global average human life expectancy was 73 years in 2023 [9]. Despite these differences, pre-clinical research efforts using these animal models have enriched our understanding of the mechanistic aspects of HSC homing and engraftment, as well as the patho-physiological changes in HSCs.

## 3. Mechanism of HSC Homing: From Tail Vein to Bone Marrow

The process of HSC translocation from the vein (usually the tail vein in mice and one of the central veins in humans) to the bone marrow (BM) involves a series of intricate steps that are essential for the successful engraftment of transplanted cells. Upon infusion into the bloodstream, HSCs must navigate through the circulatory system and home to the BM niche, where they can proliferate and differentiate to reconstitute the hematopoietic system [10]. The fate of the infused HSCs is significantly influenced by the BM microenvironment, which consists of niches. Interactions between HSCs and non-stem-cell neighbors are crucial for maintaining the quiescent state or promoting self-renewal and proliferation, but a precise understanding of the complex network of signals remains an active area of research. Following HSC transplantation, several critical events occur within the recipient’s body. These include homing and lodgment of transplanted HSCs within the BM niche, the initiation of immune reconstitution and the process of inducing immune tolerance (incomplete immune tolerance can manifest as graft rejection or a graft-versus-host response), and the early phases of hematopoietic recovery with the early emergence of donor-derived neutrophils and platelets [11], which arise primarily from short-term progenitors, and are followed by waves of hematopoietic regeneration derived from HSCs. The BM niche is not a uniform environment, and HSCs are distributed within distinct anatomical locations. HSCs preferentially localize to specific regions within the BM, such as the endosteal surface, perivascular areas, and sinusoidal vessels [12]. The endosteal niche, which is in close proximity to bone surfaces, is characterized by interactions with mesenchymal stromal cells (MSCs) in relative hypoxia, osteoblastic cells, and osteoblast-derived factors, such as CXCL12, stem-cell factor (SCF), and angiopoietin-1. In contrast, the perivascular niche surrounding sinusoidal vessels is associated with interactions with endothelial cells and pericytes and factors like CXCL12, SCF, and Notch ligands [13]. Notch signaling in HSCs enhances megakaryocyte production and platelet formation through Dll1 ligand, while Notch2 signaling through Jagged-1 generates short-term progenitor cells and long-term HSCs post-myeloablation, hindering myeloid differentiation [14,15]. Studies have shown that perivascular cells expressing Lepr and nestin+ reticular cells, as well as NG2+ pericytes, are associated with the regulation of HSC quiescence and proliferation [16,17]. Moreover, recent studies demonstrated that osteoblasts can expand hematopoietic progenitors in vitro, suggesting that genetic or chemical targeting of osteoblasts also impacts HSC numbers within the BM [18]. It has been proposed that osteoblasts interact with HSCs via the N-cadherin receptors. Activated osteoblasts can produce osteopontin, angiopoietin-1, and thrombopoietin, which limit HSC expansion and contribute to HSC quiescence [19]. Furthermore, osteoclasts can maintain HSCs and localize them to the endosteal region via bone resorption and calcium release. CXCL12-abundant reticular cells predominantly contact HSCs near sinusoids in endosteal and non-endosteal marrow [20]. In addition to the cellular components of the BM niche, the sympathetic nervous system can regulate HSC mobilization via the release of noradrenaline in the BM, which regulates CXCL12 expression in the BM. Taken together, the vascular niche plays a very important role in HSC localization and maintenance.

## 4. The Role of the Bone-Marrow Niche in HSC Homing

Upon reaching the BM, the HSCs adhere to the endothelium of the BM vasculature, extravasate, and migrate to the BM stroma. The initial period of hematopoietic recovery is characterized by gradual reconstitution of donor-derived hematopoiesis with the emergence of progeny from the abundant short-term progenitors, which have limited proliferative capacity. Hematopoiesis emerges in waves with a dynamic process that yields cells derived from increasingly rare progenitors with greater differentiation capacity, ending with everlasting multilineage hematopoiesis derived from HSCs with robust self-renewal capacity.

The homing of HSCs to the bone-marrow stem-cell niches is a critical step towards successful engraftment of HSCs post transplantation. The engraftment process requires coordination between multiple chemokine and cytokine signaling pathways and cell adhesion molecules. E-endothelial and P-endothelial selectins are essential for cell movement in BM micro-vessels. Intercellular adhesion molecule-1 (ICAM-1) and vascular cell adhesion molecule-1 (VCAM-1) play key roles in HSC homing [21,22], with α4β1/VLA-4 integrin and lectins playing primary roles in HSC attachment to marrow stromal cells [23,24]. Expression of the stromal-cell-derived factor-1 (SDF-1) ligand [25,26], also known as CXC chemokine ligand (CXCL)12, has been found to be increased in the BM niche following the conditioning regimens prior to HSC transplantation. CXCL12 is a chemokine expressed by osteoblasts and endothelial cells, and is a subset of reticular cells in the osteoblast and vascular niches of the BM. CXCL12 binds to CXCR4 (G-protein-coupled receptor) to drive HSC dormancy and cell survival [27]. Activation of the CXCR4 receptor by CXCL12 has been one of the most studied signaling axes in recent years owing to its importance in HSC mobilization to and from the BM. The interaction between CXCRL12 and CXCR4 triggers chemotaxis via intracellular GTPase proteins, which are downregulated and ubiquitinated by E3 ubiquitin ligase [28]. Extracellular nucleotides (eNTPs) such as adenosine triphosphate (ATP), uridine triphosphate (UTP) [29], and sphingosine-1-phosphate (S1P) [30] act as chemotactic factors in modulating HSC migration in the presence of CXCRL12. Pre-treatment with eUTP has significantly enhanced HSC homing to the BM. The eNTPs act through P2 nucleotide receptors (P2Rs), particularly P2YRs, which activate their signal transduction pathways via phospholipase C or adenylate cyclase [29,31]. Although the influence of CXCL12 on HSC mobilization is well-documented, its role in initial homing is not well-characterized. The evidence suggests that HSCs can migrate to the BM independently of the CXCL12–CXCR4 axis. HSC homing in a murine model refractory to CXCL12 by incubation and co-injection with AMD3100 (now also called ‘plerixafor’, a CXCR4 receptor antagonist) showed normal or only slightly reduced BM cellularity [32,33].

Successfully engrafted cells can have various degrees of potential in their abilities of renewal and differentiation, as well as varying preferences for localization within specific BM niche compartments. Several studies have combined fluorescence-activated cell sorting (FACS) and xenotransplantation assays to demonstrate the multilineage repopulation potential of the various hematopoietic stem and progenitor cell populations. Long-term stem cells (LT-HSCs) are a rare population of cells that differentiate into short-term HSCs (ST-HSCs) or lineage-restricted progenitors. The progenitor cells then ultimately undergo proliferation and differentiation to give rise to terminally differentiated cells [34]. Both ST-HSCs and LT-HSCs home to the bone-marrow microenvironment, where they self-renew and differentiate as needed [35]. Tracking the transplanted cells and their progenies with the murine system using bromo-deoxyuridine (BrdU) can provide valuable information on the homing capabilities of ST-HSC and LT-HSC, as well as regarding their differentiation dynamics. BrdU can be incorporated into the DNA of replicating cells during the S phase of the cell cycle, and its detection provides a sensitive measure of cell division, even in rare cell populations like HSCs. HSCs, defined as Ki-67− or BrdU label-retaining Lin-CD150+CD48-CD41− cells, are primarily located within the arterioles of the endosteal region, surrounded by the NG2 (CSPG4+) pericytes. Upon activation, the HSCs move away from the NG2+ cells within the periarteriolar niche to the Lepr-expressing perisinusoidal niche [36]. Cycling HSCs are often found within the sinusoids of the bone. However, it remains unclear how the bone influences HSC distribution [12,37].

## 5. Measurements of Engraftment in Mouse Models

Measuring the engraftment status requires assessment of multiple criteria, as during HSCT, not only the HSCs, but also the progenitors and mature cells are transfused. The progenitors and mature cells ensure rapid reconstitution of the recipient’s hematopoietic system as the HSCs home in the bone-marrow niche, prior to going through the engraftment process of differentiated blood cell formation over the many weeks and months that follow. The more-differentiated progenitors engraft quickly and provide initial short-term hematopoiesis, limited to their respective lineages, in the early days to weeks following myeloablation. ST-HSCs lack the ability to produce detectable GM progeny past 6–12 weeks, followed by other more differentiated cells such as multipotent progenitors (MPPs), common myeloid progenitors (CMPs), and common lymphoid progenitors (CLPs) [38]. Mouse models have been pivotal in understanding and distinguishing between short- and long-term engraftment of HSCs, and the phenotype of the cells involved in each. In a recent study, transplantation of Lin-CD34+CD38^lo^CD36− cells in NOD-SCID mice was shown to successfully engraft within 2 weeks and generate myelo-erythoid cells. This class of HSCs can maintain the hematopoietic system in the short-term (usually 8–12 weeks) [39]. Similarly, NSG mice have been used to define LT-HSCs; CD34^+^CD38-CD45RA-Thy1^+^CD49f^+^ cells were found to maintain hematopoiesis for 16+ weeks [34,35]. Also, different methods have been developed to purify HSCs and isolate them into subpopulations that are easier to quantify based on their differentiation potentials. Purified adult-mouse BM cells that yield >25% lymphomyeloid repopulating cells were defined as LT-HSCs, based on their ability to produce more than 1% of circulating WBCs for 16 weeks [40,41]. As observed, different intrinsic mechanisms are involved, which results in direct and indirect impacts on the engraftment potential. Gradual accumulation of different mutations in HSC clones is one such factor that impacts the self-renewal and fate-decision potentials of the HSCs.

## 6. Clonal Hematopoiesis

HSCs accumulate different somatic mutations with the aging of the individual. Once sufficient mutations have been accumulated, certain HSC clones acquire an advantage over others in producing progeny. Thus, the overrepresentation of mature blood cells from the same HSC clone leads to clonal hematopoiesis (CH) [42]. Prevalence of CH is low (~1%) in individuals under 50 years of age; however, it increases 10-fold after the age of 65 [43]. If the CH persists without any clinical consequences, it is referred to as clonal hematopoiesis of indeterminate potential (CHIP) [44,45]. Transcription epigenetic regulatory genes such as DNA methyltransferase enzyme DNMT3A and DNA demethylase TET2, as well as the polycomb group protein transcriptional repressor ASXL1, are among the genes which have been found to be mutated in the individuals with CH [46,47,48]. Many factors play a role in the likelihood of accumulating these mutations, including the size of the clone, the number of mutations, and the gene affected. These mutations have biases to subsets of HSCs, namely, lymphoid-biased, balanced, and myeloid-biased HSCs, as categorized by Cho et al., in which clonal composition analysis in the context of aging demonstrated loss of lymphoid-biased HSCs and hyper-prevalence of myeloid-biased HSCs [49]. This shift in HSC composition may be related to the functions of the HSCs impaired with aging. Thus, understanding the complex biology of normal HSCs and the progressive changes that give rise to clonal hematopoiesis and the various activities of HSPC pre- and post-transplantation could provide clarity on the biology of HSCs.

Developmental progression of hematopoietic cells depends on the functional contributions of key genes, with an intrinsic regulation of short- and long-term renewal involving distinct processes. Studies have shown a consistency in the repopulation behavior of clonally amplified cells in both in vivo and in vitro clones. These clones exhibit a high degree of similarity, suggesting a preset mechanism driving the changes, which requires further exploration [50]. Barcoded vector libraries and retroviral integration sites can be utilized in animal models to better track the HSPC clones upon transplantation into animal models [51]. Recently, selective expansion of CRISPR-edited adult and umbilical-cord-blood-derived CD34+ cell clones was demonstrated in murine models when ASXL1 function was lost [52]. These findings add to the knowledge of the complex regulation of HSC biology that contributes to CH development.

**Figure 1:**
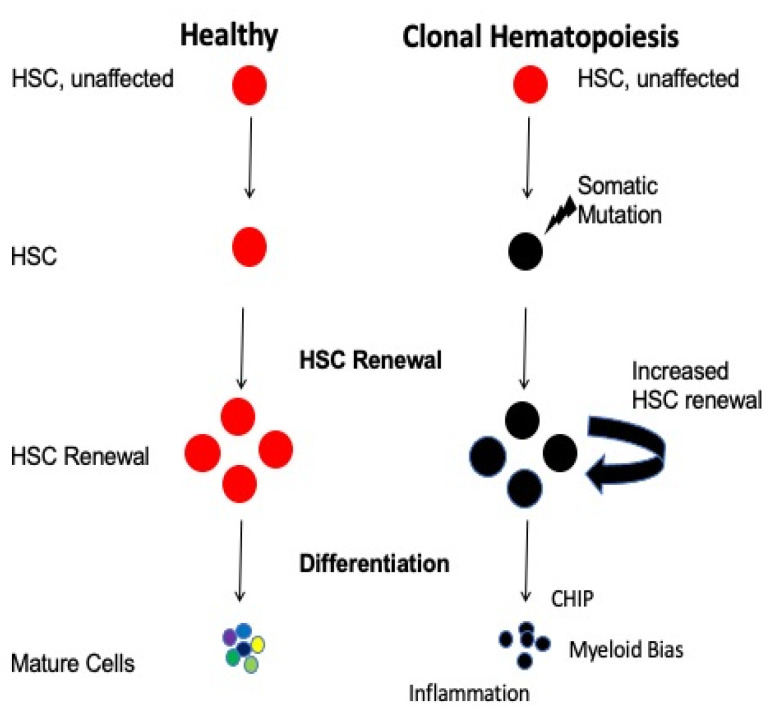
**Schematic representation of clonal hematopoiesis.** Somatic mutations caused as people age give certain clones of HSCs a competitive advantage over other clones, thereby leading to their taking over the hematopoietic system.

CH has a wide range of physiological effects and has been identified as correlating with illnesses such as cardiovascular disease (CVD), due to its correlation with increases in inflammatory states and susceptibility to coronary calcification, and therefore, a higher rate of atherosclerosis in both human and mice [53] (Figure 1). This makes CH a useful biomarker for identifying various risk factors for diseases. Given these considerations regarding the multitude of patho-physiological changes accumulated in HSCs with aging, younger donors could be preferable to aged donors, in the context of HCT, to maximize the engraftment potential, hematopoietic system reconstitution, and improvement of the BM microenvironment in a competitive manner in the recipient [54].

## 7. Competitive Repopulation

**Figure 2:**
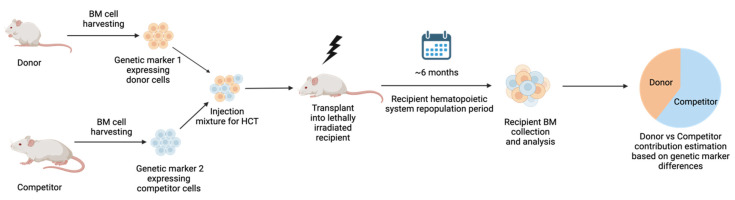
**Schematic representation of competitive repopulation assay to estimate the engraftment and hematopoietic system repopulation capacity of donor vs. competitor HSCs.** Donor and competitor BM cells are distinguished by unique genetic markers. Donor and competitor cells are then mixed and transplanted into lethally irradiated recipient mice. Following approximately 6 months of repopulation period, recipient BM is harvested to assess cellular composition and the contribution of donor origin vs. competitor origin, based on selected genetic markers. Figure created with BioRender.com (accessed on 15 December 2023).

Healthy HSCs compete for space and resources and thus ensure their engraftment in the BM niche following transplantation (Figure 2), as evidenced by nonmyeloablated murine studies [55,56,57]. Notably, though, a recent study using CD45.1 and CD45.2 mice provides evidence of empty niche locations which allow transplanted HSCs to engraft without directly competing with HSCs already established in the niche [58]. Regardless of niche space availability, CXCL12 and SCF secreted by CXCL12 abundant reticular cells and endothelial cells, respectively, help encourage the transplanted HSCs to engraft in the niche in a competitive manner [27,59]. This physiological balance is disrupted in leukemia, in which leukemic stem cells (LSCs) start releasing increased amounts of G-CSF, IL-1α, MIP-1β, and MIP-2, along with a reduction in CXCL12 expression [60]. Moreover, leukemic myeloid cells can stimulate the MSCs to overproduce osteoblastic lineage cells (OBC) which have reduced expression of HSC retention factors like Lepr, CXCL12, N-cadherin, SCF, ANGPT1, and SLIT2. These OBCs simultaneously downregulate quiescence-enforcing TGFB1 while upregulating myeloid-promoting TGFB2 [61]. The combination of these changes in the LSCs and BM niche environment creates a hostile environment for normal HSCs, while providing the LSCs a competitive advantage. Therefore, successful treatment of leukemia, as well as relapse prevention, require improved engraftment of healthy HSCs as well as reversal of the LSC-altered BM niche environment. Healthy HSCs rely on the optimum BM microenvironment condition for effective homing and engraftment. Therefore, different pre- and post-transplantation interventions can be considered to improve these aspects of HCT. For instance, co-infusion of MSCs and injections of G-CSF and GM-CSF, as well as N-acetyl-L-cysteine (NAC), have been shown to restore the health of the BM microenvironment [62,63,64]. These strategies may enhance the competitive repopulation capacity of the donor cells. Improving the competitive repopulation capacity of the transplanted HSCs can improve the patient outcome when using HCT in the treatment of different diseases.

## 8. Long-Term Engraftment and Its Influence on Leukemia Relapse

Despite recent advancements in leukemia treatment protocols, relapse of the disease remains, to date, a major cause of patient mortality [65,66,67]. Relapse is caused by a combination of factors resulting from LSC properties as well as the altered BM niche environment. Cellular signaling pathways like Wnt/β-catenin are activated by upregulation of FOXM1, which leads to β-catenin stabilization, and therefore the quiescence of LSCs [68]. On the other hand, activation of the hedgehog pathway in leukemia can accelerate the disease pathogenesis [69]. Similarly, the BM niche also contributes to maintaining LSCs through CXCL12 by MSCs. CXCL12 has been shown to be responsible for LSC quiescence as well as resistance to tyrosine kinase inhibitor treatment [70]. Given the quiescent and treatment-resistant nature of LSCs, one strategy to treat leukemia and minimize the chance of subsequent relapse would be to completely deplete the BM niche of LSCs and then perform a high-dose HSCT. Plerixafor’s ability to competitively bind to CXCR4 can be leveraged to displace the LSCs from the BM niche with this strategy. Moreover, plerixafor treatment has been shown to increase chemosensitivity of these LSCs [71,72]. This treatment, in combination with established chemo- and radiotherapies, has the potential to deplete the BM niche of the refractory/quiescent LSCs. Subsequent high-dose HSCT then can repopulate the BM niche with healthy HSCs by long-term engraftment and reduce the chance of disease relapse. Strategies like these that aim to remodel the pathological state of the BM niche could be worthwhile to explore in the future in order to develop robust treatments of hematological malignancies while improving engraftment.

## 9. Strategies to Improve Engraftment

Over the last two decades, the overall 3-year mortality rate of the recipients of HCT has been about 50%, due to various factors related to either the donor or the recipient [73]. Therefore, in the explorations aiming to improve engraftment success, studies have investigated various avenues, including usage of better HSC sources, optimization of the stem-cell numbers through mobilization procedures, and the identification and enhancement of different signaling pathways that are involved in engraftment. Different sources of HSPCs for transplant have different engraftment potentials, as well as different advantages and disadvantages associated with them. For example, cells sourced from BM have a higher engraftment potential compared to the ones from umbilical cord blood (CB), as BM source contains a higher HSPC dosage. However, finding rare types of human leukocyte antigen (HLA) in a matched donor can be a limiting factor for the BM source. Using CB as a source in these instances can be a good alternative, with the added benefit of decreased incidence of GVHD [74,75]. Another source of HSCs is peripheral blood. HSC mobilizers like G-CSF are used to increase the HSC numbers in peripheral blood. Moreover, combining G-CSF with CXCR4 blockers like plerixafor to has been proven to increase mobilization efficiency [76,77]. These strategies can mobilize a dosage of cells sufficient for successful HCT. Cell sorting is another method to consider in this context, as it allows the infusion of an HSC-enriched population, which may provide better engraftment in the long-term. Moreover, HSCs are rare populations, and sufficient quantities are difficult to obtain. Therefore, ex vivo platforms using a combination of small molecules in a synergistic manner have been shown to improve the HSC expansion and overcome these limitations [78]. In this context, the route of delivery of the cells for HCT is of critical importance. Opting for direct injection of the HSPCs in the BM can bypass some of the elements of the homing process and thereby improve engraftment efficiency, even where there is a low cell dosage from the donor [79]. Another strategy to improve engraftment is the use of gene editing techniques like CRISPR-Cas9. For example, the HBB, HBG1/2, and BCL11A genes for sickle cell disease and the CCR5 gene for acquired immunodeficiency syndrome have been tested in this context. Gene-edited hematopoietic stem and progenitor cells (HSPCs) have been found to engraft earlier than non-edited ones. However, persistence of the gene-edited HSPCs in the recipient needs to be improved for this strategy to be used it as a standard treatment protocol in clinic [80].

Furthermore, the identification of the different signaling pathways that are involved in engraftment provides an understanding of how it can be enhanced. The mechanisms involving leukocyte migration and adhesion used by LT-HSC and ST-HSC are the ligand for E-selectin, sailyl Lewis-X (sLex), and the receptor for CXCL12, CXCR4 chemokine receptor [35]. CXCL12 belongs to the α-chemokines, which function as chemo attractants for both committed and primitive hematopoietic progenitors, and a decrease in its level was shown to delay engraftment [11]. In addition, multiple studies have focused on factors that lead to stem-cell aging, in order to better understand factors that limit engraftment and that may contribute to poor graft function after transplant. For instance, cyclin-dependent kinase inhibitor p16^INK4a^ expression has been found to increase with aging. Notably, p16^Ink4a^-deficient aged HSC demonstrated rejuvenation in cell-cycle activity and engraftment capacity [81]. Other studies have reported a regulatory role of p19^ARF^ in p53 stability through the inactivation of the ubiquitin ligase Mdm2. Moreover, mice overexpressing truncated isoforms of p53 (DNp53) or the short isoform of p53 (p44) exhibited accelerated aging and reduced HSC engraftment capabilities [82,83]. Furthermore, novel strategies like collecting the donor cells in hypoxic condition prior to transplant [84], as well as treatments of donor cells with short-term hyperthermia [85] have been shown to improve engraftment in murine studies. Knowledge of these intra- and extracellular factors gives a better understanding of how the HSC fate is regulated, and thus can help optimize HCT strategies to improve engraftment. Table 1 provides a comprehensive list of different factors/molecules, the study models, and the impact of the listed factor/molecules on engraftment.

## 10. Conclusions

In this review we have explored different xeno-transplantation models in HCT research, as well as HSC homing, which allows HSCs to reach the bone-marrow microenvironment, engraft, and proliferate. The anatomy and the various cellular components of the BM niche play pivotal roles in ensuring efficient engraftment and maintenance of the transfused HSCs. In this context, various animal models are used to ask and understand the important questions about HSC biology. While these xeno-transplantation models have advanced our understanding of HSC and HCT, there are fundamental differences with humans in terms of their genetic make-up and life spans. Therefore, interpreting these pre-clinical research data in a clinical context requires consideration of these differences. Despite these shortcomings, the pre-clinical animal studies provide a better understanding of clinically relevant aspects of HCT and engraftment, such as CH and competitive repopulation. Finally, we have highlighted the current research and potential strategies for improving the HCT success rate. These strategies include, but are not limited to, the use of various HSC sources with different engraftment potentials, ex vivo expansion platforms, modifications of different chemokines that alter HSC engraftment capabilities, and the novel CRISPR-Cas9 gene-editing technique. The knowledge from the pre-clinical studies continues to clarify the complex interplay of different factors affecting HCT and improve the safety and efficacy of this widely used treatment modality.

## Figures and Tables

**Table 1:** List of factors/molecules that influence HSC engraftment and the animal models used to study these factors/molecules.

Engraftment Factors	Function	Study Model	Influence on Engraftment	References
Stromal-cell-derived factor-1 (SDF-1) also known as CXCL12	Chemokine isolated from stromal fibroblasts and abundantly expressed in BM.	NOD/LtSz-scid/scid (NOD/SCID) mice and MxCre-CXCR4^f/null^ mice and C57BL/6	Actuates and promotes HSC maintenance and improves engraftment	Lapidot, T. 2005 [25] Plett, P. et al. 2002 [27] Onai, N. et al. 2000 [33]
Notch ligands	Signal through Jagged-1 generates short-term progenitor cells and long-term HSCs post-myeloablation, hindering myeloid differentiation	Transgenic Mice: Mx-Cre+ × ROSA^DNMAML/+^ mice; C57BL/6 (B6, CD45.2^+^); and (B6-SJL, CD45.1^+^)	Supports HSC self-renewal and improves engraftment	Varnum-Finney, B. et al. 2011 [14] Maillard, I. et al. 2008 [15]
Lepr and nestin + reticular cells	Associated with the regulation of HSC quiescence and proliferation	Transgenic Mice: Tie2-cre and leptin receptor (LepR)-cre mice and Col1-caPPR mice	Improves HSC frequency in the bone marrow	Xiao, Y. et al. 2022 [16] Boulais, P. E. et al. 2015 [17]
N-cadherin	Osteoblast direct interactions via N-cadherin-mediated adhesion support HSC function	Transgenic mice: Scl-tTA::TRE-BCR/ABL (BA) double-transgenic mouse—CML	Positively regulates HSCs in BM niche	Hosokawa, K et al. 2010 [19] Schepers, K et al. 2013 [61]
Osteopontin, angiopoietin-1, and thrombopoietin	Activated osteoblasts can produce osteopontin, angiopoietin-1, and thrombopoietin, which limit HSC expansion and contribute to HSC quiescence	Transgenic mice: Mx1-Cre+Bmpr1a^fx^ mutant mice	Shown to positively impact HSC regulation	Hosokawa, K. et al. 2010 [19]
Intercellular adhesion molecule-1 (ICAM-1)	Plays a role in homing through mediating cellular adhesion interaction	Transgenic mice: C57BL/6 and 129S strains P/E−/− (C57/Bl6J × 129S) Mice lacking the two selectins (P and E−)	Positively regulates HSCs in BM niche	Frenette, P. S. et al. 1998 [21]
Vascular cell adhesion molecule-1 (VCAM-1)	Plays a role in homing through mediating rolling and firm adhesion of HPC in BM	Transgenic mice: C57/Bl6J × 129S P/E−/−	Positively regulates HSCs in BM niche	Mazo, I. B. et al. 1998 [22]
α4β1/VLA-4 integrin and lectins	Primary roles in HSC attachment to marrow stromal cells	NOD/SCID	HSC homing by enabling attachment to the vascular endothelium	Peled et al. 2000 [24]
Adenosine triphosphate (ATP) and uridine triphosphate (UTP)	Extracellular nucleotide (eNTPs) act as potent chemotactic factors in modulating HSC migration in the presence of CXCL12	NOD/SCID	UTP and ATP (to a lesser extent) modulate HSC motility and homing to BM niche	Rossi, L. et al. 2007 [29]
Sphingosine-1-phosphate (S1P)	Extracellular nucleotide (eNTPs) act as potent chemotactic factors in modulating HSC migration in the presence of CXCL12	Transgenic: B6.Cg-Tg(UBC-cre/ERT2)1Ejb/J	Homing of HSPC	Adamiak, M. et al. 2015 [30]
N-acetyl-L-cysteine (NAC)	Shown to restore the health of BM microenvironment	NOD/SCID and NSG mice	Increase in human HSC engraftment and multilineage hematopoietic differentiation	Hu, L. et al. 2014 [64]
TGF-B1, TGF-B2, and SLIT2	TGF-B2 promotes myeloid differentiation and TGF-B1/SLIT2 are HSC retention factors, all support HSC function	BCR/ABL (BA) mice	Regulate quiescence and self-renewal of HSCs	Schepers, K et al. 2013 [61]

BM, Bone marrow; HSC, Hematopoietic stem cell; CXC-Chemokine ligand 12 (CXCL12)-abundant reticular cells; LepR + cells, Leptin receptor (LepR)-positive cells; NG2+ cells, Neural–glial antigen 2 (NG2)-positive cells; SCF, Stem-cell factor; CXCL12, C-X-C motif chemokine ligand 1; Angpt-1, Angiopoietin 1; Vcam1, Vascular cell adhesion protein 1; VLA-4, Very late antigen-4; TGF-β1/2, Transforming growth factor beta 1/2.
